# Comparative analysis of adverse events and flares across COVID-19 vaccine platforms in SLE patients

**DOI:** 10.1097/MD.0000000000047509

**Published:** 2026-01-30

**Authors:** Worawit Louthrenoo, Supparat Konsamun, Punsita Tangkum, Wanitcha Gumtorntip, Nuntana Kasitanon, Poramed Winichakoon, Antika Wongthanee

**Affiliations:** a Department of Internal Medicine, Division of Rheumatology, Faculty of Medicine, Chiang Mai University, Chiang Mai, Thailand; b Department of Internal Medicine, Research Unit, Faculty of Medicine, Chiang Mai University, Chiang Mai, Thailand; c Department of Internal Medicine, Division of Infectious Diseases and Tropical Medicine, Faculty of Medicine, Chiang Mai University, Chiang Mai, Thailand.

**Keywords:** adverse events, COVID-19 vaccine, flares, systemic lupus erythematosus

## Abstract

Direct comparison of adverse events (AEs) and flares among the coronavirus disease 2019 (COVID-19) vaccine platforms in systemic lupus erythematosus (SLE) patients has rarely been determined. This study aimed to compare AEs and flares among 3 different COVID-19 vaccine platforms in SLE patients. Risk factors for SLE flares following vaccination were also determined. Adult SLE patients completing 2 doses of primary series COVID-19 vaccine (inactivated virus, adenovirus-vectored, and mRNA vaccines), were included in this study. The patients were followed up until 3 months after the 2nd vaccine dose or study censor. SLE disease activity and flares were determined by Systemic Lupus Erythematosus Disease Activity Index-2000 (SLEDAI-2K) and Safety of Estrogens in Lupus Erythematosus National Assessment – SLEDAI Flare Index, but the modified SLEDAI-2K score was used instead of the original SLEDAI-2K. Sixty-seven, 19, and 46 patients received inactivated virus, adenovirus-vectored, and mRNA vaccines, respectively. The incidence rate and 95% confidence interval (IR [95% CI])/100 patient-months was not different for overall AEs (38.2 [27.6–53.0], 44.2 [23.8–82.2], and 49.3 [35.7–68.0], respectively, P = .496) and flares (10.4 [6.1–17.5], 14.6 [6.1–35.2], and 7.1 [3.8–13.3], respectively, *P* = .561). Severe flares were more common than mild-to-moderate ones, primarily due to renal flare, regardless of vaccine types. There was no significant change in SLE disease activity after vaccination across the vaccine types. High number of the 1997 American College of Rheumatology classification criteria and use of combination immunosuppressive drugs at 1st vaccine dose were independent risk factors for flares. There was no significant difference in overall AEs, changes in SLE disease activity, frequency, and severity of flares, regardless of the vaccine platforms.

## 1. Introduction

During the coronavirus disease 2019 (COVID-19) pandemic, several COVID-19 vaccines from different platforms were developed, and in response, several countries were authorized to use them in emergency circumstances.^[1]^ However, despite effectively reducing morbidity and mortality from COVID-19 infection, they have been associated with a significant number of adverse events (AEs).^[2]^

Systemic lupus erythematosus (SLE) is an autoimmune disease characterized by remission and relapse. The pathogenesis of SLE is not clearly understood, but activation of the innate and adaptive immunity has been involved.^[3]^ Patients with SLE are at increased risk of infection, not only from an aberrance in the immune system, but also corticosteroid and immunosuppressive drug treatment.^[4]^ SLE patients clearly have a greater risk than the general population of acquiring COVID-19 infection, rate of severe disease, hospitalization, and death.^[5]^

During the COVID-19 pandemic, the Thai government and health organizations provided COVID-19 vaccines to the population. Three types of vaccines were available: inactivated virus vaccines (CoronaVac [Sinovac Biotech, China] and BBIBP-CorV or Covilo [Sinopharm, China]), 2) adenovirus-vectored vaccine (AZD1222 or Covishield [AstraZeneca, United Kingdom]), and 3) mRNA vaccines (BNT162b2 or Comirnaty [Pfizer-BioNTech, USA] and mRNA-1273 or COVID-19 Moderna [Moderna, USA]). The inactivated virus vaccines were available 1st, followed by the other 2 types a few months later. Individuals who wanted the vaccine had to make an appointment and indicate their preferred vaccine type using the “Moh-Prompt” application (a government-provided app.) on their smartphones and reported any AEs on days 1 to 7 and 8 to 30 after each vaccine dose. Due to the limited supply and availability of some vaccines, everyone could receive either a single type or mixed schedule (mixed type of vaccine). We previously reported AEs and flares in patients with SLE.^[6,7]^ In those studies, we found no significant differences in AEs or flares among the different types or doses of COVID-19 vaccines. Since the patients were allowed to receive mixed types of vaccines, it was not known whether the lack of significant differences in AEs and flares was attributable to the heterogeneous effects of the mixed vaccine regimens.

A recent systematic review reported AE rates and flare rates after COVID-19 vaccination in SLE patients of 12% to 92% and 0% to 20%, respectively.^[8]^ From screening this systematic review, the rates of AEs and flares seemed more frequent following mRNA vaccination than after the other ones. Clearly, the mRNA vaccine has shown higher immunogenicity and produced higher antibody response levels compared to other vaccine types.^[9,10]^ However, whether these factors contributed to higher rates of AEs and flares remains unclear. Interestingly, a direct comparison of AEs and flares among the COVID-19 vaccine types has rarely been determined.^[11]^

Therefore, this study aimed to compare the AEs and flares among different types of COVID-19 vaccine platforms in SLE patients who had completed the primary vaccine series (2 doses). The AEs and flares between the 1st and 2nd dose of the same vaccine were compared. In addition, possible risk factors for the development of SLE flares following COVID-19 vaccination were also determined.

## 2. Materials and methods

### 2.1. Patients and data collection

The data of this study were derived from those in previous studies reported by the authors.^[6,7]^ In brief, consecutive SLE patients aged ≥20 years from the Chiang Mai University Lupus Cohort who completed 2 doses of the same COVID-19 vaccine and attended the rheumatology clinic between March and October 2022 were included. Patients with overlapping rheumatic diseases, a history of COVID-19 infection prior to the 1st vaccine dose, or those who had COVID-19 infection after the 1st vaccine dose up to 90 days after the 2nd vaccine dose or study censor were excluded. Demographics, clinical features, laboratory findings, and treatment received by the patients were obtained from the medical records from 6 months prior to the 1st vaccine dose until the next visit (or up to 3 months) after the 2nd dose or study censor. Because vaccination and clinic appointment dates differed, disease activity on the vaccination date was determined using clinical and laboratory data from the last visit before vaccination. Patients with significant post-vaccination symptoms were usually asked to return for assessment, where disease activity and flares were also determined. Patients were excluded if they developed COVID-19 infection during the study. In addition, AEs were collected from patients and also from their “Moh-Prompt” smartphone application. The patients were asked to rate the severity of the AEs: symptoms that were self-manageable and did not interfere with activities of daily living were considered mild or moderate, whereas those that interfered with activities of daily living or required a physician visit were considered severe. Furthermore, the patients were asked whether they thought the symptoms after vaccination were AEs or disease flares. Patient self-report of flares was based on the patient’s own judgment; however, final flare classification was determined by physician assessment.

### 2.2. Classification criteria and assessment tools

SLE was diagnosed according to either the 1997 American College of Rheumatology (ACR) Updating Criteria for Classification of Systemic Lupus Erythematosus,^[12]^ or the 2012 Systemic Lupus Erythematosus International Collaboration Clinics classification criteria for SLE.^[13]^ SLE disease activity was determined by the modified Systemic Lupus Erythematosus Disease Activity Index-2000 (mSLEDAI-2K).^[14]^ Severity of SLE disease activity was classified according to Abrahamowicz et al,^[15]^ but using the mSLEDAI-2K score instead of the original SLEDAI-2K (remission, mild, moderate, high, and very high for the mSLEDAI-2K scores of 0, 1–5, 6–10, 11–19, and ≥20, respectively). SLE flares and degree of flares were determined by the Safety of Estrogens in Lupus Erythematosus National Assessment (SELENA) Flare Index, but using the mSLEDAI-2K score instead of the original SLEDAI-2K score.^[16]^ Organ damage was determined by the Systemic Lupus Erythematosus International Collaboration Clinics/ACR damage index.^[17]^

### 2.3. Ethical approval

This study was approved by the Ethic Committee of the Faculty of Medicine, Chiang Mai University (No. 107/2022, date March 22, 2022). All of the participants provided their written informed consent prior to entering the study.

### 2.4. Statistical analysis

The statistical analysis was performed using STATA 16.0 for Windows (Stata Corporation, Texas). Continuous variables were presented as mean ± standard deviation or median (p25–p75), where appropriate. Categorical variables were described as percentages. To compare differences between 2 independent groups, the Student *t* test and Mann–Whitney *U* test were used for continuous data with normal and non-normal distribution, respectively. The Chi-square test and Fisher exact test were used for categorical data as appropriate. To compare differences between the 3 groups, the 1-way analysis of variance and Kruskal–Wallis test were used for continuous data with normal and non-normal distribution, respectively, and logistic regression for binary data. The paired *t* test and sign-rank test were used to determine the change of continuous data with normal and non-normal distribution, respectively, and the McNemar test was used to determine the change in proportion. Poisson regression was used to compare the incidence rates. Multivariable analysis was performed using Cox regression. For the zero-cell count, Cox Regression with Firth Penalized likelihood was performed. Fisher protected least significant difference or Dunn test was used for pairwise comparisons. A *P*-value of <.05 was considered statistically significant.

## 3. Results

### 3.1. Demographic and characteristics of patients studied

From the 201 patients in the previous study, 67, 19, and 46 of them completed 2 doses of inactivated virus, adenovirus-vectored, and mRNA vaccines, respectively (Table 1). None of them consumed alcohol or smoked. Overall, there were no significant differences in the clinical characteristics between patients in these 3 vaccine groups in terms of gender, age, SLE disease duration, comorbidities, cumulative manifestations of the 1997 ACR SLE classification criteria, current organ manifestations at time of the 1st vaccine dose, organ damage, and SLE disease activity (mSLEDAI-2K score) at 6 and 3 months prior to and at the 1st vaccine dose. The proportion of patients who received prednisolone was higher in the inactivated virus group; however, this difference was not statistically significant (*P* = .059). In contrast, patients in the mRNA group received a higher median daily dose of prednisolone (*P* = .047; mRNA group vs inactivated virus group, *P* = .005). The mean duration of observation was significantly different between the 3 groups (*P* < .001), that being the mRNA and adenovirus-vectored groups had significantly longer duration than the inactivated virus group (*P* = .001, and *P* = .036, respectively).

**Table 1:** Baseline characteristics of patients at the 1st COVID-19 vaccine dose.

Baseline characteristics	Type of vaccines			
	Inactivated virus (N = 67)	Adenovirus-vectored (N = 19)	mRNA (N = 46)	*P*-value
Female	67 (100.0)	18 (94.7)	45 (97.8)	.525
Age (yr)	41.4 ± 11.3	45.1 ± 15.5	43.8 ± 14.8	.467
SLE disease duration (yr)	13.5 (7.1–19.1)	12.8 (7.3–23.9)	12.8 (4.2–21.5)	.926
Comorbidity*				
Hypertension	18 (26.9)	6 (31.6)	14 (30.4)	.881
Dyslipidemia	27 (40.3)	5 (26.3)	14 (30.4)	.394
Thyroid disease	6 (9.0)	4 (21.1)	3 (6.5)	.217
Diabetes mellitus	4 (6.0)	2 (10.5)	2 (4.4)	.649
Previous malignancy	1 (1.5)	1 (5.3)	1 (2.2)	.651
Hepatitis B or C infection	1 (1.5)	0	1 (2.2)	.789
Others	1 (1.5)†	0	2 (4.4)	.376
Stroke	1 (1.5)	0	1 (2.2)	.789
Depression	0	0	1 (2.17)	–
NTM	1 (1.5)	0	0	–
Cumulative 1997 ACR manifestations				
Malar rash	34 (50.8)	8 (42.1)	28 (60.9)	.339
Oral/nasal ulcers	17 (25.4)	6 (31.6)	14 (30.4)	.785
Photosensitivity	13 (19.4)	3 (15.8)	4 (8.7)	.312
Discoid rash	15 (22.4)	5 (26.3)	19 (41.3)	.096
Arthritis	40 (59.7)	13 (68.4)	30 (65.2)	.724
Serositis	10 (14.9)	2 (10.5)	6 (13.0)	.877
Neurological disorder	3 (4.5)	1 (5.3)	2 (4.4)	.986
Renal involvement	54 (80.6)	14 (73.7)	33 (71.7)	.528
Hematologic disorder	50 (74.6)	18 (94.7)	37 (80.4)	.212
Anti-nuclear antibody (ever)	67 (100.0)	19 (100.0)	46 (100.0)	–
Immunologic, n/N (%)				
Anti-dsDNA antibody (ever)	51/64 (79.7)	13/16 (81.3)	31/40 (77.5)	.931
Anti-Sm antibody (ever)	2/6 (33.3)	2/2 (100.0)	5/9 (55.6)	.581
Anti-phospholipid antibodies‡ (ever)	5/36 (13.9)	0	3/24 (12.5)	.758
Number of 1997 ACR classification criteria	5.3 ± 1.2	5.5 ± 1.2	5.6 ± 1.2	.622
SLICC/ACR damage index	0.7 ± 1.0 0 (0–1)	0.6 ± 1.0 0 (0–2)	1.1 ± 1.6 1 (0–2)	.144
Current active organ involvement				
Renal system	23 (34.3)	8 (42.1)	9 (19.6)	.126
Mucocutaneous system	10 (14.9)	1 (5.3)	6 (13.0)	.568
Musculoskeletal system	1 (1.5)	0	1 (2.2)	.789
Hematological system	1 (1.5)	1 (5.3)	1 (2.2)	.651
Gastrointestinal system	1 (1.5)	0	1 (2.2)	.789
Neurological system	0	0	0	–
SLE disease activity (mSLEDAI-2K)				
6 months prior to 1st vaccination date	2.3 ± 3.4 0 (0–4)	3.0 ± 3.1 4 (0–4)	1.9 ± 2.9 0 (0–4)	.436
3 months prior to 1st vaccination date	2.1 ± 3.6 0 (0–4)	3.1 ± 3.5 4 (0–4)	1.6 ± 2.6 0 (0–4)	.252
1st vaccination date	2.1 ± 3.6 0 (0–4)	3.0 ± 3.4 4 (0–4)	1.5 ± 2.5 0 (0–4)	.264
Baseline medications				
Prednisolone	63 (94.0)	14 (73.7)	39 (84.8)	.059
Dose (mg/day)	5 (5–7.5)	5 (5–7.5)	5 (5–15)	.047 (.005)∥
Hydroxychloroquine	18 (26.9)	6 (31.6)	18 (39.1)	.392
Immunosuppressive drugs	36 (53.7)	9 (47.4)	22 (47.8)	.786
Mycophenolate mofetil	31 (46.3)	9 (47.4)	15 (32.6)	.306
Azathioprine	3 (4.5)	0	5 (10.9)	.207
Methotrexate	2 (3.0)	0	2 (4.4)	.702
Cyclophosphamide	0	0	1 (2.2)	–
Calcineurin inhibitor§	7 (10.5)	4 (21.1)	2 (4.4)	
Temporary discontinuation immunosuppressive drugs				
Prior to vaccination	5 (7.5)	2 (10.5)	3 (6.5)	.858
After vaccination	5 (7.5)	1 (5.3)	1 (2.2)	.507

Data are expressed as mean ± SD, median [p25–p75], or n (%), n/N = number of positive tests/number of patients tested.

Continuous data with normal and non-normal distribution were analyzed by 1-way analysis of variance (ANOVA) and the Kruskal–Wallis test, respectively. Pairwise comparisons were analyzed by Dunn test. Binary data were analyzed by logistic regression.

ACR = American College of Rheumatology, mSLEDAI-2K = modified Systemic Lupus Erythematosus Disease Activity Index-2000, NTM = non-tuberculous mycobacterium infection, SLE = systemic lupus erythematosus, SLICC = Systemic Lupus Erythematosus International Collaboration Clinics.

* 1 patient might have more than 1 comorbid disease.

† 1 patient had stoke and NTM infection.

‡ Any anti-cardiolipin antibody, lupus anti-coagulant, or beta-2-glycoprotein-1 antibody.

§ Cyclosporine or tacrolimus.

∥ *P*-value between mRNA vaccine vs inactivated virus vaccine.

### 3.2. Adverse events

The overall AEs among the 3 vaccine types ranged from 53.7% to 80.4% (Table 2), which was more commonly observed in the mRNA group (*P* = .016; mRNA group vs inactivated virus group, *P* = .002). However, the incidence rate (IR) with 95% confidence interval (95% CI) was not different among the 3 groups. Local symptoms were also more frequent in the mRNA group (*P* < .001; mRNA group vs inactivated virus group, *P* < .001); while constitutional symptoms occurred more frequently in the adenovirus-vectored 1 (*P* = .014; adenovirus-vectored group vs inactivated virus group, *P* < .004). The AEs were most frequent in the musculoskeletal, neurological, and mucocutaneous systems (13.4–26.3%, 13.4–26.3%, and 1.5–15.8%, respectively). Although organ-based AEs were not different among the vaccines, they were more frequent in the adenovirus-vectored group. Interestingly, no AEs in the hematological system were observed.

**Table 2:** Adverse events following 2 doses of primary series of COVID-19 vaccine.

Adverse events	Type of vaccine			*P*-value
	Inactivated virus (N = 67)	Adenovirus-vectored (N = 19)	mRNA (N = 46)	
Duration of observation in days†	41.0 (26.0–56.5)	56.5 (36.5–64.5)	57.75 (48.0–73.5)	<.001 (.001)* (.036)**
IR (95% CI) of adverse events/100 patient-months	38.2 (27.6–53.0)	44.2 (23.8–82.2)	49.3 (35.7–68.0)	.496
Total observation period in months	94.19	22.60	75.10	
Overall adverse events‡	36 (53.7)	13 (68.4)	37 (80.4)	.016 (.002)*
Local symptoms	20 (29.9)	10 (52.6)	30 (69.6)	<.001 (<.001)*
Constitutional symptoms	15 (22.4)	11 (57.9)	17 (37.0)	.014 (.004)**
Musculoskeletal system	9 (13.4)	5 (26.3)	7 (15.2)	.407
Gastrointestinal system	1 (1.5)	0	4 (8.7)	.105
Mucocutaneous system	1 (1.5)	3 (15.8)	4 (8.7)	.106
Hematological system	0	0	0	–
Neurological system	9 (13.4)	5 (26.3)	8 (17.4)	.418
Cardiopulmonary system	1 (1.5)	0	1 (2.2)	.789
Severity of adverse events rated by patients, n/N (%)				
Mild-to-moderate§	33/67 (49.3)	11/19 (57.9)	33/44 (75.0)	.030 (.004)*
Severe‖	3/67 (4.5)	2/19 (10.5)	2/44 (4.6)	.581
Adverse events requiring physician visits, n/N (%)	1/67 (1.5)	3/19 (15.8)	2/46 (4.4)	.078
Patients’ rating of flares after vaccination (within 30 days)	6 (9.00)	2 (10.5)	3 (6.5)	.650

Data are expressed as n (%). n/N = number of positive responses/number of respondents. IR (95% CI) = incidence rate (95% confidence interval).

Binary data were analyzed by logistic regression. The incidence rate was compared by Poisson regression.

Local symptoms: pain, redness, or swelling at the injection site. Constitutional symptoms: fever, malaise, headache, or tiredness. Musculoskeletal system: muscle ache and pain, muscle weakness, or joint pain. Gastrointestinal system: nausea, vomiting, or diarrhea. Mucocutaneous system: rashes, hair loss, or itching. Hematological symptoms: bruising, ecchymosis, or spot bleeding on skin. Neurological system: drowsiness, sleepiness, numbness, or dizziness. Cardiopulmonary system: chest pain or tightness, or difficulty in breathing.

† Duration from the 1st vaccination date until the next visit (or up to 3 months) after the 2nd dose of vaccine or study censor.

‡ The same adverse events occurring after the 1st and 2nd vaccine dose were counted as 1 event.

§ As self-manageable or not interfering with activity of daily living (ADL).

‖ Interfering with ADL or needing physician visits.

* *P*-value comparing between mRNA vaccine vs inactivated virus vaccine.

** *P*-value comparing between adenovirus-vectored vaccine vs inactivated virus vaccine.

The severity of AEs rated by the patients was considered mild-to-moderate at 49.3% to 75.0%, which was more frequent in the mRNA group (*P* = .030; mRNA group vs inactivated virus group, *P* = .004). The severe AEs ranged from 4.5% to 10.5%, which showed no difference between vaccine types. AEs requiring physician visits occurred at 1.5% to 15.8%, with a more frequent tendency in the adenovirus-vectored group (*P* = .078). Overall, 6.5% to 10.5% of the patients perceived SLE flares as being caused by the vaccines, which was more frequent in the adenovirus-vectored group.

Adverse events occurring after the 1st and 2nd vaccine doses were compared (Table S1, Supplemental Digital Content, https://links.lww.com/MD/R287). Overall, IRs (95% CIs) tended to be lower after the 2nd dose in the inactivated virus and adenovirus-vectored groups and higher in the mRNA group; none of these differences reached statistical significance. There were no statistically significant differences between doses in local or constitutional symptoms, organ-specific AEs, patient-rated severity, or AEs requiring physician visits across vaccine types.

### 3.3. Changes in SLE disease activity and flares

SLE disease activity, flares, and organ flares are shown in Table 3. No difference in SLE disease activity (mSLEDAI-2K score) was observed at baseline (1st vaccine dose) among the 3 types of vaccine. Disease activity was also not different on the last observation date. Similarly, no significant difference was observed in SLE disease activity between the 1st vaccination date and the last observation date, regardless of vaccine types. Furthermore, although the frequency (total, mild-to-moderate or severe), and IR (95% CI) of flares were not different regardless of the vaccine types, they tended to be more frequent in the adenovirus-vectored group. Renal, mucocutaneous and hematologic systems were the most common organ flares, with no significant difference between the vaccine types.

**Table 3:** SLE disease activity, flares, and organ-specific flares after 2 doses of primary series of COVID-19 vaccine.

SLE disease activity and flares	Type of vaccine			*P*-value*
	Inactivated virus (N = 67)	Adenovirus-vectored (N = 19)	mRNA (N = 46)	
Duration of observation in days†	41.0 (26.0–56.5)	56.5 (36.5–64.5)	57.75 (48.0–73.5)	<.001 (.001)** (.036)***
IR (95% CI) of flares/100 patient-months	10.4 (6.1–17.5)	14.6 (6.1–35.2)	7.1 (3.8–13.3)	.561
Total observation period in months	135.20	34.17	140.29	
SLE disease activity (mSLEDAI-2K score)				
1st vaccination date	2.1 ± 3.6 0 (0–4)	3.0 ± 3.4 4 (0–4)	1.5 ± 2.5 0 (0–4)	.377
Last observation date‡	2.3 ± 3.6 0 (0–4)	2.1 ± 2.2 2 (0–4)	1.9 ± 2.9 0 (0–4)	.761
*P*-value****	.358	.218	.148	
Total flares (events)	17 (25.4)	7 (36.8)	13 (28.3)	.611
Mild-to-moderate	8 (11.9)	3 (15.8)	5 (10.9)	.858
Severe	9 (13.4)	4 (21.1)	8 (17.4)	.358
Organ flares				
Mucocutaneous system (alopecia, skin rash, vasculitis rash)	3 (4.5)	2 (10.5)	4 (8.7)	.551
Hematological system (autoimmune hemolysis, leukopenia, thrombocytopenia)	2 (3.0)	2 (10.5)	1 (2.2)	.301
Musculoskeletal system (myositis, arthritis)	1 (1.5)	0	1 (2.2)	.789
Renal system (nephritis, proteinuria)	10 (14.9)	3 (15.8)	6 (13.0)	.704
Neurological system (central and peripheral nervous system)	0	0	1 (2.17)	-
Gastrointestinal system	0	0	0	–
Constitutional symptoms (fever, malaise, weight loss)	1 (1.5)	0	0	–

Data are expressed as mean ± SD, median (p25–p75), or n (%). IR (95% CI) = incidence rate (95% confidence interval).

SLEDAI-2K was analyzed by 1-way analysis of variance (ANOVA). Flares were analyzed by logistic regression. The incidence rate of flares was analyzed by Poisson regression.

mSLEDAI-2K = modified Systemic Lupus Erythematosus Disease Activity Index-2000, SLE = systemic lupus erythematosus.

† Duration from the 1st vaccination date until the next visit (or up to 3 months) after the 2nd dose of vaccine or study censor.

‡ next visit or up to 3 months after the 2nd vaccine dose or study censor.

* *P*-value = *P*-value comparing between the 3 types of vaccine.

** *P*-value comparing between mRNA vaccine vs inactivated virus vaccine.

*** *P*-value comparing between adenovirus-vectored vaccine vs inactivated virus vaccine.

**** *P*-value = *P*-value comparing mSLEDAI-2K score between the 1st vaccination date and last observation date.

A sub-analysis was performed to compare SLE disease activity, flares, and organ-specific flares between the 1st and 2nd doses (Table S2, Supplemental Digital Content, https://links.lww.com/MD/R287). The observation period was longer after the 2nd dose across vaccine groups. SLE disease activity (mSLEDAI-2K score) did not differ between the 1st and 2nd doses within any vaccine type at either the vaccination date or the last observation date. Within each vaccine type, changes in mSLEDAI-2K from the vaccination date to the last observation date after either dose were not statistically significant. Total flares were more frequent after the 2nd dose, that reached statistical significance only in the mRNA group (*P* = .034), and mild-to-moderate flares were significantly higher after the 2nd dose in the inactivated virus and mRNA groups (*P* = .034 and *P* = .025, respectively). However, the IRs (95% CIs) of flares did not differ between the 1st and 2nd doses across vaccine types. Renal, mucocutaneous, and hematologic flares were most common; only mucocutaneous flares were higher after the 2nd dose than the 1st dose in the mRNA group (*P* = .046).

### 3.4. Predictors of flares after vaccination

Due to the small number of flares occurring in each vaccine type, cases with and without flares were combined, resulting in 24 and 108 cases with and without flares, respectively (Table 4). Overall, the clinical characteristics including gender, age, SLE disease duration, and comorbidities were not different between those with and without flares. However, patients with flares had significantly more photosensitivity (*P* = .006), anti-dsDNA (*P* = .042), and mean number of the 1997 ACR classification criteria (*P* < .001). They also had higher SLE disease activity (mSLEDAI-2K score) at 6 months prior to the 1st vaccine dose (*P* < .001), but not 3 months prior to or at the 1st vaccine dose. At the 1st dose, patients with flares had more mucocutaneous and gastrointestinal involvement than those without (*P* = .001 and .032, respectively). In terms of treatment received, no significant difference in the use of prednisolone (including median daily dose), and anti-malarial and immunosuppressive drugs was observed between those with and without flares; however, those with them received more calcineurin inhibitors and combination immunosuppressive drugs (*P* = .006 and .011, respectively).

**Table 4:** Baseline characteristics of patients between those with and without flares after 2 doses of primary series of COVID-19 vaccine.

Baseline characteristics	Cases with flares (N = 24)	Cases without flare (N = 108)	*P*-value
Female	23 (95.8)	107 (99.1)	.332
Age (yr)	42.1 ± 10.4	42.9 ± 13.8	.775
SLE disease duration (yr)	13.8 (7.9–17.1)	12.8 (6.7–21.3)	.972
Type of vaccine			
Inactivated virus	11 (45.8)	56 (51.9)	.859
Adenovirus-vectored	4 (16.7)	15 (13.9)	
mRNA	9 (37.5)	37 (34.3)	
Comorbidity*			
Hypertension	10 (41.7)	28 (25.9)	.139
Dyslipidemia	8 (33.3)	38 (35.2)	.863
Thyroid disease	2 (8.3)	11 (10.2)	.999
Diabetes mellitus	2 (8.3)	6 (5.6)	.637
Previous malignancy	1 (4.2)	2 (1.9)	.455
Hepatitis B or C infection	1 (4.2)	1 (0.9)	.332
Others	0	3 (2.8)†	.999
Cumulative 1997 ACR manifestations			
Malar rash	16 (66.7)	54 (50.0)	.139
Oral/nasal ulcers	9 (37.5)	28 (25.9)	.253
Photosensitivity	8 (33.3)	12 (11.1)	.006
Discoid rash	11 (45.8)	28 (25.9)	.053
Arthritis	14 (58.3)	69 (63.9)	.610
Serositis	3 (12.5)	15 (13.9)	.999
Neurological disorder	1 (4.2)	5 (4.6)	.999
Renal involvement	20 (83.3)	81 (75.0)	.595
Hematologic disorder	19 (79.2)	86 (79.6)	.959
Anti-nuclear antibody (ever)	24 (100.0)	108 (100.0)	-
Immunologic, n/N (%)			
Anti-dsDNA antibody (ever)	22/23 (95.7)	73/97 (75.3)	.042
Anti-Sm antibody (ever)	1/4 (25.0)	8/13 (61.5)	.294
Anti-phospholipid antibodies‡ (ever)	2/15 (13.3)	6/52 (11.5)	.999
Number of 1997 ACR classification criteria	6.2 ± 1.4	5.3 ± 1.1	<.001
SLICC/ACR damage index	1.3 ± 1.8 0.5 (0–2)	0.7 ± 1.0 0 (0–1)	.124 .177
Current active organ involvement			
Renal system	9 (37.5)	31 (28.7)	.396
Mucocutaneous system	68 (33.3)	9 (8.3)	.001
Musculoskeletal system	0	2 (1.9)	.999
Hematological system	2 (8.3)	1 (0.9)	.085
Gastrointestinal system	2 (8.3)	0	.032
Neurological system (central and peripheral nervous system)	0	0	-
SLE disease activity (mSLEDAI-2K score)			
6 months prior to 1st vaccination date	4.4 ± 3.7 4 (1–7.5)	1.7 ± 2.8 0 (0–4)	<.001
3 months prior to 1st vaccination date	2.8 ± 3.2 2 (0–4)	1.9 ± 3.3 0 (0–4)	.200
1st vaccination date	2.8 ± 3.2 2 (0–4)	1.8 ± 3.2 0 (0–4)	.162
Baseline medications			
Prednisolone	22 (91.7)	94 (87.0)	.735
Dose (mg/day)	5 (5–10)	5 (5–10)	.529
Hydroxychloroquine	5 (20.8)	37 (34.3)	.201
Immunosuppressive drugs	14 (58.3)	53 (49.1)	.412
Mycophenolate mofetil	12 (50.0)	43 (39.8)	.360
Azathioprine	2 (8.3)	6 (5.6)	.637
Methotrexate	0	4 (3.7)	.999
Cyclophosphamide	0	1 (0.9)	.999
Calcineurin inhibitor§	6 (25.0)	7 (6.5)	.006
Combination immunosuppressive drugs	6 (25.0)	8 (7.4)	.011

Data are expressed as mean ± SD, median [p25–p75], or n (%), n/N = number of positive tests/number of patients tested.

Continuous data with normal and non-normal distribution were analyzed by the Student *t* test and Mann–Whitney *U* test, respectively. Chi-square test and Fisher exact test were used for categorical data as appropriate.

ACR = American College of Rheumatology, mSLEDAI-2K = modified Systemic Lupus Erythematosus Disease Activity Index-2000, NTM = non-tuberculous mycobacterium infection, SLICC = Systemic Lupus Erythematosus International Collaboration Clinics.

* 1 patient might have more than 1 comorbid disease.

† Stroke in 2, NTM infection in 1, and depression in 1 (1 patient had stroke and NTM infection).

‡ Anti-cardiolipin antibody, lupus anti-coagulant, or beta-2-glycoprotein-1 antibody.

§ Cyclosporine or tacrolimus.

Clinically relevant variables and those with *P*-value ≤ .15 in the univariable analyses were included in the multivariable logistic regression analysis. Two models of logistic regression analyses were determined, due to the multicollinearity between mSLEDAI-2K scores and organ involvement (renal, musculoskeletal, hematological, and neurological systems) (Table 5). In Model 1 (mSLEDAI-2K scores not included), risk factors for the development of flares were number of 1997 ACR classification criteria (hazard ratio with 95% CI (HR [95% CI]): 1.92 [1.28–2.88, *P* = .002), current hematological involvement (HR [95% CI] 15.97 [2.93–86.95], *P* = .001), current gastrointestinal involvement (HR [95% CI] 14.16 [2.05–97.92], *P* = .007), and combination immunosuppressive drugs use (HR [95% CI] 11.82 [3.31–42.18], *P* < .001). In Model 2 (mSLEDAI-2K scores included), SLE disease duration (HR [95% CI] 0.92 [0.85–0.99], *P* = .026), number of 1997 ACR classification criteria (HR [95% CI] 1.82 [1.27–2.62], *P* = .001), and combination immunosuppressive drug use were risk factors for the development of flares (HR [95% CI] 8.55 [2.63–27.84], *P* < .001).

**Table 5:** Predicting factors for flares after 2 doses of primary series of COVID-19 vaccine.

Baseline characteristic	Total N = 132	Flare (%) N = 24	Univariable			Multivariable					
						Model 1 (mSLEDAI-2K not included)			Model 2 (mSLEDAI-2K and GI included)		
			HR	95% CI	*P*-value	HR	95% CI	*P*-value	HR	95% CI	*P*-value
Female	130	23 (17.7)	.32	.04–2.45	.275	.42	.04–4.13	.454	.41	.04–4.08	.445
Age (yr)	132	24 (18.2)	.99	.96–1.02	.472	1.03	.98–1.07	.274	1.01	.97–1.06	.545
SLE disease duration (yr)	132	24 (18.2)	.99	.94–1.03	.526	.94	.87–1.01	.091	.92	.85–0.99	.026
Type of vaccine											
Inactivated virus	67	11 (16.4)	Ref.								
Adenovirus-vectored	19	4 (21.1)	1.54	.48–4.97	.469						
mRNA	46	9 (19.6)	.76	.31–1.85	.541						
Number of 1997 ACR classification criteria	132	24 (18.2)	1.48	1.09–2.00	.013	1.92	1.28–2.88	.002	1.82	1.27–2.62	.001
SLICC/ACR damage index	132	24 (18.2)	1.21	.96–1.52	.109				1.33	.93–1.92	.123
Current active organ involvement*											
Renal system	40	9 (22.5)	1.94	.84–4.51	.122						
Mucocutaneous system	17	8 (47.1)	3.03	1.28–7.17	.011	2.68	.94–7.66	.066			
Musculoskeletal system	2	0	2.63	.02–19.72	.559						
Hematological system	3	2 (66.7)	4.55	1.05–19.67	.043	15.97	2.93–86.95	.001			
Gastrointestinal system	2	2 (100.0)	4.14	.95–18.04	.059	14.16	2.05–97.92	.007	7.75	.81–74.13	.075
Neurological system	0	0	–	–	–						
mSLEDAI-2K score*	132	24 (18.2)	1.08	.98–1.20	.129						
Baseline medications*											
Prednisolone	116	22 (19.0)	2.15	.50–9.19	.303						
Hydroxychloroquine	42	5 (11.9)	.57	.21–1.53	.263						
Immunosuppressive drugs	67	14 (20.9)	1.99	.84–4.71	.115						
Combination immunosuppressive drugs	14	6 (42.9)	3.35	1.31–8.58	.012	11.82	3.31–42.18	<.001	8.55	2.63–27.84	<.001

Multivariable analysis was analyzed by Cox regression. HR = hazard ratio, 95% CI = 95% confidence interval.

ACR = American College of Rheumatology, GI = gastrointestinal system, mSLEDAI-2K = modified Systemic Lupus Erythematosus Disease Activity Index-2000, SLICC = Systemic Lupus Erythematosus International Collaboration Clinics.

* At time of the 1st vaccine dose.

The relationship between baseline SLE disease activity and flares also was determined. No difference in flare frequency between SLE patients with remission or those with low (mSLEDAI-2K score ≤ 6), and those with moderate or higher disease activity (mSLEDAI-2K > 6) was seen regardless of the vaccine type (data not shown).

## 4. Discussion

This study found that AEs and flares were commonly observed (54–80% and 25–37%, respectively) across the 3 vaccine groups. The frequency of overall AEs was significantly higher in the mRNA group; however, the IR (95% CI) of overall AEs was not different. Local symptoms were more frequent in the mRNA group, whereas constitutional symptoms were more frequent in the adenovirus-vectored group. Mild-to-moderate AEs, as reported by the patients, were more frequent in the mRNA group, but severe AEs or those requiring physician visits did not differ between the 3 groups. There was no difference in the frequency (total, mild-to-moderate, or severe) and IR (95% CI) of flares across vaccine types. The renal, mucocutaneous, and hematologic systems were the most common organ flares. Severe flares were more common than mild-to-moderate flares across vaccine types, driven by renal flares. Long disease duration, a higher number of the 1997 ACR classification criteria, hematologic and gastrointestinal involvement, and use of combination immunosuppressive drugs at the 1st vaccine dose were associated with a higher risk of flares.

In order to compare the AEs and flares in this study to those previously reported, studies in adult SLE patients who had completed 2 doses of COVID-19 vaccine were reviewed and are shown in Table S3, Supplemental Digital Content, https://links.lww.com/MD/R287. Of these, mRNA vaccines were the most commonly reported,^[11,18–26]^ followed by inactivated virus vaccines.^[11,27–31]^ The adenovirus-vectored vaccines have rarely been reported.^[27]^ There was great variation in the frequency of AEs and flares reported. For example, overall AEs in the inactivated virus group were reported at 25%,^[30]^ compared with 44% to 100% from the mRNA group.^[18,19,21,24]^ Local reactions were reported to be as low as 1.7% in the inactivated virus group,^[30]^ whereas there were 39.5% to 48.5% in the mRNA group.^[18,19,21,24]^ Fatigue or tiredness, headache, and joint and muscle pain were the most commonly reported systemic AEs, and they were usually mild in severity. The frequency of all flares was reported as 4.7% to 19.4%,^[28,31]^ in the inactivated virus group and 2.4% to 10.4%^[20,22,24]^ in the mRNA group. These flares were generally mild-to-moderate (10.0–25.7%), with severe flares occurring in 1.3% to 8.3%.^[20,21,25]^ Joints and skin were the most commonly involved organs. Only 1 study directly compared AEs and flares between inactivated virus and mRNA vaccines. Interestingly, although many patients showed improvement in their SLEDAI-2K scores, anti-dsDNA levels, and degree of proteinuria, none required an increase in medication after vaccination.^[11]^ In this study, the frequencies of AEs (overall, local, and systemic) from the inactivated virus and mRNA vaccines were also similar to those previously reported. However, this study found a higher frequency of all flares, with severe flares being more common than mild-to-moderate flares. The high frequency of severe flares in this study might be due to the majority of patients having renal flares (76.5% of them had a history of nephritis, and 30.3% had active renal involvement at the time of the 1st vaccine dose). These patients developed new-onset or increased 24-hour proteinuria (>0.5 g protein/g creatinine), and required an increased medication dose or additional medications, thus fulfilling the definition of severe flares.^[16]^

Several studies have compared AEs and flares between the 1st and 2nd vaccine doses, mostly involving mRNA vaccines, and have showed conflicting results (Table S3, Supplemental Digital Content, https://links.lww.com/MD/R287). For the inactivated virus vaccine, some studies found that overall AEs were lower after the 2nd dose than after the 1st,^[29,31]^ whereas others found the opposite.^[11]^ For the mRNA vaccine, some studies indicated that the frequency of overall AEs was lower after the 2nd dose,^[11]^ while others reported frequencies similar to those after the 1st.^[26]^ The frequency of flares after the 1st and 2nd doses was reported as 8% and 13%, respectively, for inactivated virus vaccine,^[28]^ and 9% and 20%, respectively, for mRNA vaccine.^[26]^ In this study, the frequency of AEs after the 2nd dose was lower in the inactivated virus group and higher in the mRNA group, whereas the frequency of flares was higher after the 2nd dose in both the inactivated virus and mRNA groups. These variations in the reported frequencies of AEs and flares might be due to differences in study design (prospective, retrospective, or cross-sectional), how AEs were categorized (e.g., overall AEs, local AEs, and systemic or organ-based AEs), the timing of AE and flare assessments after vaccination, and the instruments used to determine flares (SELENA-SLEDAI Flare Index, BILAG-2004, or physician judgement).

Although most studies, including the present study, found a notable number of flares, no significant change in SLE disease activity occurred after vaccination, regardless of vaccine type (Table S3, Supplemental Digital Content, https://links.lww.com/MD/R287). A report from Japan using the mRNA vaccine found a modest but statistically significant increase in the SLEDAI score after the 2nd vaccine dose, accompanied by a 14% flare frequency.^[21]^ The reason for no significant increase in SLE disease activity, despite a notable number of flares, might be due to the small proportion of patients with flares post-vaccination, which were usually mild-to-moderate. Therefore, the increase in SLEDAI score would not be large enough to make a significant difference when compared with the value at baseline or at the 1st dose. In addition, flares occurring in an organ with preexisting disease activity at the time of the 1st vaccination (e.g., increasing rashes, alopecia, number of arthritic joints, degree of proteinuria, etc) would have the same score post-vaccination; however, flares were captured when a new medication was added, the medication dosage was increased, or by other definitions of flares.^[16]^ The notable number of severe flares (mainly in the renal system) without a significant increase in mSLEDAI-2K scores observed in this study may be explained by the fact that 30% of patients had some active renal involvement at the 1st vaccine dose, resulting in no further increase in the mSLEDAI-2K score. Interestingly, a certain number of patients were reported who had improved clinically or decreased disease activity after mRNA vaccination.^[11,24]^

Although flares in SLE patients after COVID-19 vaccination have been reported in many studies, studies focusing on risk or associated factors remain limited.^[21,26]^ A study from Japan found that high SLEDAI scores and anti-dsDNA antibody levels were associated with flares.^[21]^ Another 1 from Peru found that a history of renal involvement and hydroxychloroquine use reduced the risk of flares, whereas azathioprine increased it.^[26]^ The findings that a high mean cumulative number of the 1997 ACR classification criteria (which might be related to longer disease duration), hematologic and gastrointestinal involvement, and the use of a combination of immunosuppressive drugs at the 1st vaccine dose were risk factors for flares in this study are different from those previously reported. However, the large HRs with wide 95% CIs for hematologic and gastrointestinal involvement as risk factors for flares after vaccination suggest potential overfitting due to the small number of cases and should be interpreted with caution. Furthermore, the risk factors for flares in this study differed from those in a previous report by our group,^[7]^ which found that renal and mucocutaneous involvement, as well as high SLE activity before the 1st vaccine dose, were independent predictors of flares. The difference might be that, in the previous report, the analysis was based on vaccine type, with most patients receiving mixed vaccine types, while this study included only those who received 2 doses of the same vaccine. Thus, the results in this study minimized the potential confounding effects of mixed vaccines on AEs and flares. Risk factors for flares in this study also differed from those observed in Japan^[21]^ and Peru,^[26]^ which may reflect differences in ethnicity and follow-up duration. Furthermore, only the mRNA vaccine was used in those 2 studies, whereas the current study assessed flares among patients who received any vaccine type. These differences should be considered when interpreting the results.

It should be noted that the prevalence of adenovirus-vectored COVID-19 vaccine use was only 1.6% to 6.5% among European SLE patients.^[32,33]^ This low prevalence may be related to concerns about thrombosis, as an increased risk has been observed in individuals receiving adenovirus-vectored COVID-19 vaccines.^[34,35]^ In contrast, this study found a relatively high prevalence of 14%, and an even higher prevalence was reported in another study from Thailand.^[27]^ This could be attributed to the unavailability or shortage of mRNA vaccines during that period, which led individuals who wanted to be vaccinated to register for either inactivated virus or adenovirus-vectored vaccines instead. Fortunately, no thrombotic episodes were reported among patients who received the adenovirus-vectored vaccine in this study.

Despite AEs and flares occurring in a measurable proportion of patients after COVID-19 vaccination, these events were usually mild-to-moderate and well tolerated, supporting the overall safety of COVID-19 vaccination in SLE patients, including those receiving immunosuppressive therapy. Before widespread vaccination, SLE patients had higher risks of COVID-19 infection, severe disease, hospitalization, and mortality than the general population. In contrast, among vaccinated SLE cohorts, rates of severe COVID-19, hospitalization, and death have been similar to those in the general population.^[5,36]^ Accordingly, SLE patients should be encouraged to receive COVID-19 vaccination and recommended boosters in line with public health guidance to reduce the risk of severe outcomes.^[37,38]^

This study had several limitations. Its retrospective nature clearly influenced the accuracy of AEs recalled by the patients. However, the “Moh-Prompt” application recorded AEs shortly after vaccination and could reduce errors and minimize missing data. The small number of patients, particularly in the adenovirus-vectored group, resulted in a rather wide 95% CI for the IR of AEs and flares. The small number of patients in the adenovirus-vectored group reflects real-world conditions in that this type of vaccine was rarely used among SLE patients (as discussed above). Given patients’ preferences and vaccine availability during the study period, this could also have led to potential selection bias and may have affected the statistical analysis. Therefore, this should be taken into consideration when interpreting the results. However, the numbers of patients in the other 2 groups were generally similar to those reported in other studies. Use of the mSLEDAI-2K instrument (which excludes anti-dsDNA antibodies and complement levels) may underestimate SLE disease activity and flare frequency compared with the original SLEDAI-2K, particularly when flares are driven mainly by serologic changes. For example, a patient with normal anti-dsDNA and complement levels at baseline who later develops elevated anti-dsDNA and low complement after vaccination this could be considered a “minor flare” according to the SELENA-SLEDAI Flare Index in a study using the original SLEDAI‑2K, but it would be classified as “no flare” in a study using the mSLEDAI‑2K (as in this study). Nonetheless, we applied the mSLEDAI-2K consistently across all 3 vaccine groups, so internal comparisons of disease activity and flares remain valid. Direct comparisons with studies that used the original instrument are therefore limited. However, the mSLEDAI-2K has demonstrated a very strong correlation with the original SLEDAI-2K, *r* = .924,^[14,39]^ which supports partial comparability across studies. In addition, due to the vaccination date and assessment date being different, and the clinical data from the last visit prior to vaccination used to assess SLE disease activity, SLE disease activity at the time of assessment might not be entirely accurate, particularly in cases where minor flares occurred and were resolved before assessment. Furthermore, this study followed patients for only 90 days after the 2nd vaccine dose, therefore, it may not capture AEs or delayed immune-mediated flares that develop later in the post-vaccination course. As the 3-month window was applied across all vaccine groups, so internal comparisons remain valid; longer-term follow-up will be required to quantify late-onset events. Lastly, this study was performed at a single center, which could limit its generalizability. More studies are needed to confirm these findings.

Despite its limitations, this study had several strengths. SLE disease activity was recorded from 6 months before the 1st vaccine dose, ensuring patient stability prior to vaccination. All of the patients in this study were in the cohort and followed regularly at 1- to 3-month intervals, during which the clinical and laboratory data were properly collected, thus ensuring reliable assessment of disease activity and flares. As the observation duration varied among the vaccine types, this study calculated the total AEs and flares using IR (95% CI) to eliminate effect on their frequency, which could provide more robust results. Lastly, this study was one of the few that compared AEs, disease activity and flares after vaccination across different vaccine platforms, which was another strength when compared to most previous reports.

## 5. Conclusion

This study found no significant differences in overall AEs, changes in SLE disease activity, or flare frequency and severity across vaccine types. It could not confirm the previously observed trend that mRNA vaccines were associated with higher frequencies of AEs and flares. Severe flares, mainly renal, were more frequent than mild-to-moderate flares across all vaccine types. High mean number of 1997 ACR criteria, and combination immunosuppressive drugs use at the 1st vaccine dose were risk factors for flares. COVID-19 vaccination is safe for patients with SLE, with AEs and flares generally mild-to-moderate and well tolerated, even among those receiving immunosuppressive therapy. SLE patients should be encouraged to receive COVID-19 vaccination and recommended boosters in accordance with public health authorities’ recommendations to achieve outcomes comparable to those in the general population.

## Acknowledgements

The authors thank Mrs Waraporn Sukitawut for her secretarial assistance.

## Author contributions

**Conceptualization:** Worawit Louthrenoo, Supparat Konsamun, Antika Wongthanee.

**Data curation:** Worawit Louthrenoo, Punsita Tangkum, Wanitcha Gumtorntip.

**Formal analysis:** Worawit Louthrenoo, Supparat Konsamun, Antika Wongthanee.

**Investigation:** Worawit Louthrenoo, Supparat Konsamun, Antika Wongthanee.

**Methodology:** Worawit Louthrenoo, Supparat Konsamun, Antika Wongthanee.

**Supervision:** Worawit Louthrenoo.

**Writing – original draft:** Worawit Louthrenoo, Supparat Konsamun.

**Writing – review & editing:** Worawit Louthrenoo, Supparat Konsamun, Punsita Tangkum, Wanitcha Gumtorntip, Nuntana Kasitanon, Poramed Winichakoon, Antika Wongthanee.

## Supplementary Material



## References

[1] Chakraborty C Bhattacharya M Dhama K. SARS-CoV-2 vaccines, vaccine development technologies, and significant efforts in vaccine development during the pandemic: the lessons learned might help to fight against the next pandemic. Vaccines (Basel). 2023;11:682. 36992266 10.3390/vaccines11030682 PMC10054865

[2] Dhamanti I Suwantika AA Adlia A Yamani LN Yakub F. Adverse reactions of COVID-19 vaccines: a scoping review of observational studies. Int J Gen Med. 2023;16:609–18. 36845341 10.2147/IJGM.S400458 PMC9951602

[3] Crow MK. Pathogenesis of systemic lupus erythematosus: risks, mechanisms and therapeutic targets. Ann Rheum Dis. 2023;82:999–1014. 36792346 10.1136/ard-2022-223741

[4] Pego-Reigosa JM Nicholson L Pooley N. The risk of infections in adult patients with systemic lupus erythematosus: systematic review and meta-analysis. Rheumatology (Oxford). 2021;60:60–72. 33099651 10.1093/rheumatology/keaa478 PMC7785308

[5] Mehta P Gasparyan AY Zimba O Kitas GD. Systemic lupus erythematosus in the light of the COVID-19 pandemic: infection, vaccination, and impact on disease management. Clin Rheumatol. 2022;41:2893–910. 35639259 10.1007/s10067-022-06227-7 PMC9152659

[6] Tangkum P Kasitanon N Gumtorntip W. COVID-19 vaccination in patients with systemic lupus erythematosus: adverse events and rating agreement of flares between patients and physicians. Int J Rheum Dis. 2024;27:e70001. 39655469 10.1111/1756-185X.70001

[7] Louthrenoo W Tangkum P Kasitanon N. Flares and predicting factors of flares in patients with systemic lupus erythematosus associated with different doses and types of COVID-19 vaccines. Vaccines (Basel). 2024;12:1399. 39772059 10.3390/vaccines12121399 PMC11728829

[8] Tan SYS Yee AM Sim JJL Lim CC. COVID-19 vaccination in systemic lupus erythematosus: a systematic review of its effectiveness, immunogenicity, flares and acceptance. Rheumatology (Oxford). 2023;62:1757–72. 36271852 10.1093/rheumatology/keac604 PMC9620290

[9] Abufares HI Oyoun Alsoud L Alqudah MAY. COVID-19 vaccines, effectiveness, and immune responses. Int J Mol Sci . 2022;23:15415. 36499742 10.3390/ijms232315415 PMC9737588

[10] Morales-Nunez JJ Munoz-Valle JF Machado-Sulbaran AC. Comparison of three different COVID-19 vaccine platforms (CoronaVac, BTN162b2, and Ad5-nCoV) in individuals with and without prior COVID-19: reactogenicity and neutralizing antibodies. Immunol Lett. 2022;251-252:20–8. 36279685 10.1016/j.imlet.2022.10.002 PMC9585342

[11] So H Li T Chan V Tam LS Chan PK. Immunogenicity and safety of inactivated and mRNA COVID-19 vaccines in patients with systemic lupus erythematosus. Ther Adv Musculoskelet Dis. 2022;14:1759720X221089586. 10.1177/1759720X221089586 PMC9021484 35464809

[12] Hochberg MC. Updating the American college of rheumatology revised criteria for the classification of systemic lupus erythematosus. Arthritis Rheum. 1997;40:1725. 10.1002/art.1780400928 9324032

[13] Petri M Orbai AM Alarcon GS. Derivation and validation of the systemic lupus international collaborating clinics classification criteria for systemic lupus erythematosus. Arthritis Rheum. 2012;64:2677–86. 22553077 10.1002/art.34473 PMC3409311

[14] Uribe AG Vila LM McGwin G Jr Sanchez ML Reveille JD Alarcon GS. The systemic lupus activity measure-revised, the mexican systemic lupus erythematosus disease activity index (SLEDAI), and a modified SLEDAI-2K are adequate instruments to measure disease activity in systemic lupus erythematosus. J Rheumatol. 2004;31:1934–40. 15468356

[15] Abrahamowicz M Fortin PR du Berger R Nayak V Neville C Liang MH. The relationship between disease activity and expert physician’s decision to start major treatment in active systemic lupus erythematosus: a decision aid for development of entry criteria for clinical trials. J Rheumatol. 1998;25:277–84. 9489819

[16] Buyon JP Petri MA Kim MY. The effect of combined estrogen and progesterone hormone replacement therapy on disease activity in systemic lupus erythematosus: a randomized trial. Ann Intern Med. 2005;142:953–62. 15968009 10.7326/0003-4819-142-12_part_1-200506210-00004

[17] Gladman DD Urowitz MB Goldsmith CH. The reliability of the systemic lupus international collaborating clinics/American college of rheumatology damage index in patients with systemic lupus erythematosus. Arthritis Rheum. 1997;40:809–13. 9153540 10.1002/art.1780400506

[18] Bartels LE Ammitzbøll C Andersen JB. Local and systemic reactogenicity of COVID-19 vaccine BNT162b2 in patients with systemic lupus erythematosus and rheumatoid arthritis. Rheumatol Int. 2021;41:1925–31. 34476603 10.1007/s00296-021-04972-7 PMC8412379

[19] Ferri C Ursini F Gragnani L. Impaired immunogenicity to COVID-19 vaccines in autoimmune systemic diseases. high prevalence of non-response in different patients’ subgroups. J Autoimmun. 2021;125:102744. 34781162 10.1016/j.jaut.2021.102744 PMC8577991

[20] Izmirly PM Kim MY Samanovic M. Evaluation of immune response and disease status in systemic lupus erythematosus patients following SARS-CoV-2 vaccination. Arthr Rheumatol. 2022;74:284–94. 34347939 10.1002/art.41937 PMC8426963

[21] Kikuchi J Kondo Y Kojima S. Risk of disease flares after SARS-CoV-2 mRNA vaccination in patients with systemic lupus erythematosus. Immunol Med. 2024;47:76–84. 38189429 10.1080/25785826.2023.2300163

[22] Ma M Santosa A Fong W. Post-mRNA vaccine flares in autoimmune inflammatory rheumatic diseases: results from the COronavirus national vaccine registry for ImmuNe diseases SINGapore (CONVIN-SING). J Autoimmun. 2023;134:102959. 36473406 10.1016/j.jaut.2022.102959 PMC9705203

[23] Mormile I Della Casa F Petraroli A. Immunogenicity and safety of mRNA anti-SARS-CoV-2 vaccines in patients with systemic lupus erythematosus. Vaccines (Basel). 2022;10:1221. 36016108 10.3390/vaccines10081221 PMC9416775

[24] Moyon Q Sterlin D Miyara M. BNT162b2 vaccine-induced humoral and cellular responses against SARS-CoV-2 variants in systemic lupus erythematosus. Ann Rheum Dis. 2022;81:575–83. 34607791 10.1136/annrheumdis-2021-221097 PMC8494536

[25] Yoshida T Tsuji H Onishi A. Medium-term impact of the SARS-CoV-2 mRNA vaccine against disease activity in patients with systemic lupus erythematosus. Lupus Sci Med. 2022;9:e000727. 35961691 10.1136/lupus-2022-000727 PMC9378947

[26] Zavala-Flores E Salcedo-Matienzo J Quiroz-Alva A Berrocal-Kasay A. Side effects and flares risk after SARS-CoV-2 vaccination in patients with systemic lupus erythematosus. Clin Rheumatol. 2022;41:1349–57. 34782941 10.1007/s10067-021-05980-5 PMC8592807

[27] Assawasaksakul T Lertussavavivat T Sathitratanacheewin S. Comparison of immunogenicity and safety of inactivated, adenovirus-vectored, and heterologous adenovirus-vectored/mRNA vaccines in patients with systemic lupus erythematosus and rheumatoid arthritis: a prospective cohort study. Vaccines (Basel). 2022;10:853. 35746461 10.3390/vaccines10060853 PMC9227480

[28] Delkash P Azimi A Taherpour N Aghajani SH. The role of sinopharm BIBP COVID-19 vaccine immunization in systemic lupus erythematous flare-up. Arch Clin Infect Dis. 2023;18:e139989.

[29] Tang Q Li F Tian J Kang J He J. Attitudes towards and safety of the SARS-CoV-2 inactivated vaccines in 188 patients with systemic lupus erythematosus: a post-vaccination cross-sectional survey. Clin Exp Med. 2023;23:457–63. 35612692 10.1007/s10238-022-00832-1 PMC9130966

[30] Wang P Ni J Chu YY. Seroprevalence of SARS-CoV-2-specific antibodies and vaccination-related adverse events in systemic lupus erythematosus and rheumatoid arthritis. Biomed Pharmacother. 2022;150:112997. 35486976 10.1016/j.biopha.2022.112997 PMC9040458

[31] Yuki EFN Borba EF Pasoto SG. Impact of distinct therapies on antibody response to SARS-CoV-2 vaccine in systemic lupus erythematosus. Arthritis Care Res (Hoboken). 2022;74:562–71. 34806342 10.1002/acr.24824 PMC9011410

[32] Gerosa M Schioppo T Argolini LM. The impact of anti-SARS-CoV-2 vaccine in patients with systemic lupus erythematosus: a multicentre cohort study. Vaccines (Basel). 2022;10:663. 35632419 10.3390/vaccines10050663 PMC9146432

[33] Larsen ES Nilsson AC Möller S Voss AB Johansen IS. Immunogenicity and risk of disease flare after a three-dose regimen with SARS-CoV-2 vaccination in patients with systemic lupus erythematosus: results from the prospective cohort study COVAC-SLE. Clin Exp Rheumatol. 2023;41:676–84. 35894059 10.55563/clinexprheumatol/b8a6zb

[34] Schultz NH Sorvoll IH Michelsen AE. Thrombosis and thrombocytopenia after ChAdOx1 nCoV-19 vaccination. N Engl J Med. 2021;384:2124–30. 33835768 10.1056/NEJMoa2104882 PMC8112568

[35] Li X Burn E Duarte-Salles T. Comparative risk of thrombosis with thrombocytopenia syndrome or thromboembolic events associated with different covid-19 vaccines: international network cohort study from five European countries and the US. BMJ. 2022;379:e071594. 36288813 10.1136/bmj-2022-071594 PMC9597610

[36] Jiang X Sparks J Wallace Z. Risk of COVID-19 among unvaccinated and vaccinated patients with systemic lupus erythematosus: a general population study. RMD Open. 2023;9:e002839. 36889799 10.1136/rmdopen-2022-002839 PMC10008206

[37] Curtis JR Johnson SR Anthony DD. American college of rheumatology guidance for COVID-19 vaccination in patients with rheumatic and musculoskeletal diseases: version 5. Arthritis Rheumatol. 2023;75:E1–E16. 36345691 10.1002/art.42372 PMC9878068

[38] Landewe RBM Kroon FPB Alunno A. EULAR recommendations for the management and vaccination of people with rheumatic and musculoskeletal diseases in the context of SARS-CoV-2: the November 2021 update. Ann Rheum Dis. 2022;81:1628–39. 35197264 10.1136/annrheumdis-2021-222006

[39] Gladman DD Ibanez D Urowitz MB. Systemic lupus erythematosus disease activity index 2000. J Rheumatol. 2002;29:288–91. 11838846

