# Supplementary material for: Comparative analysis of adverse events and flares across COVID-19 vaccine platforms in SLE patients
Source: Medicine (Baltimore). 2026 Jan 30;105(5):e47509. doi: 10.1097/MD.0000000000047509 (PMC12863779; doi:10.1097/MD.0000000000047509)
Supplement: Supplementary file 1 [file medi-105-e47509-s001.pdf]

Supplementary Table 1. Comparison of adverse events between the 2 doses of COVID-19 vaccine

	Type of vaccines								
	**Inactivated virus (N = 67)**			**Adenovirus-vectored (N = 19)**			**mRNA (N = 46)**		
	**1st dose[a]**	**2nd dose[b]**	***P*-value**	**1st dose[a]**	**2nd dose[b]**	***P*-value**	**1st dose[a]**	**2nd dose[b]**	***P*-value**
Duration of observation after vaccination in days	21 (20 – 22)	62 (31 – 86)	< .001	38 (22 – 56)	76 (30 – 96)	.025	24.5 (14 – 31)	91 (74 – 114)	< .001
IR (95% CI) of adverse events/100 patient-months	64.7 (44.1 – 95.0)	18.5 (10.0 – 34.4)	.053	59.8 (29.9 – 119.6)	21.7 (5.4 – 86.6)	.084	85.5 (59.4 – 123.0)	19.4 (9.7 – 38.9)	.158
Total observation period in months	40.18	54.01		13.37	9.23		33.94	41.17	
Overall adverse events	33 (49.3)	26 (38.8)	.052	13 (68.4)	10 (52.6)	.083	31 (67.4)	35 (76.1)	.157
Local symptoms	18 (26.9)	16 (23.9)	.414	9 (47.4)	8 (42.1)	.564	27 (58.7)	29 (63.0)	.480
Constitutional symptoms	14 (20.9)	9 (13.4)	.059	10 (52.6)	6 (31.6)	.103	9 (19.6)	15 (32.6)	.058
Musculoskeletal system	7 (10.5)	8 (11.9)	.564	5 (26.3)	3 (15.8)	.157	6 (13.0)	5 (10.9)	.564
Gastrointestinal system	1 (1.5)	1 (1.5)	.999	0	0	-	4 (8.7)	2 (4.4)	.157
Mucocutaneous system	1 (1.5)	1 (1.5)	.999	3 (15.8)	2 (10.5)	.317	3 (6.5)	2 (4.4)	.564
Hematological system	0	0	-	0	0	-	0	0	-
Neurological system	9 (13.4)	8 (11.9)	.317	5 (26.3)	3 (15.8)	.157	6 (13.0)	6 (13.0)	.999
Cardiopulmonary system	1 (1.5)	1 (1.5)	.999	0	0	-	0	1 (2.2)	.317
Severity of adverse events rated by patients, n/N (%)									
Mild-to-moderate[c]	31/67 (46.3)	24/67 (35.8)	.052	11/19 (57.9)	10/19 (52.6)	.564	28/44 (63.6)	30/43 (69.8)	.317
Severe[d]	2/67 (3.00)	2/67 (3.00)	.999	2/19 (10.5)	0	.157	1/44 (2.3)	2/43 (4.7)	.317
Adverse events requiring a physician visit, n/N (%)	1/67 (1.5)	0	.317	3/19 (15.8)	0	.083	1/46 (2.2)	1/46 (2.2)	.999

Data are expressed as n (%). n/N = number of positive responses/number of respondents. IR (95% CI) = incidence rate.

Continuous data was analyzed by sign rank test, and changes in proportion were analyzed by McNemar's test. p-value for the comparison between the 1st and 2nd doses of the vaccine

[a] = from 1st vaccine dose to 2nd vaccine dose, [b] = next visit or up to 3 months after the 2nd vaccine dose or study censor, [c] = as self-manageable or not interfering with activity of daily living (ADL), [d] = interfering with ADL or needing physician visits.

Local symptoms: pain, redness, or swelling at the injection site; Constitutional symptoms: fever, malaise, headache, or tiredness; Musculoskeletal system: muscle ache and pain, muscle weakness, or joint pain; Gastrointestinal system: nausea, vomiting, or diarrhea; Mucocutaneous system: rashes, hair loss, or itching; Hematological symptoms: bruising, ecchymosis, or spot bleeding on skin; Neurological system: drowsiness, sleepiness, numbness, or dizziness; Cardiopulmonary system: chest pain or tightness, or difficulty in breathing.

Supplementary Table 2. Comparison of SLE disease activity, flares, and organ flares between the 2 doses of COVID-19 vaccine

	**Type of vaccine**								
	**Inactivated virus (N = 67)**			**Adenovirus-vectored (N = 19)**			**mRNA (N = 46)**		
	**1st dose[a]**	**2nd dose[b]**	***P*-value***	**1st dose[a]**	**2nd dose[b]**	***P*-value***	**1st dose[a]**	**2nd dose[b]**	***P*-value***
Duration of observation after vaccination in days	21 (20 – 22)	62 (31 – 86)	< .001	38 (22 – 56)	76 (30 – 96)	.025	24.5 (14 – 31)	91 (74 – 114)	< .001
IR (95%CI) of all flares	10.7 (4.8 – 23.8)	7.6 (4.2 – 13.8)	.453	7.7 (1.9 – 30.8)	9.8 (3.7 – 26.1)	.636	7.1 (2.3 – 22.0)	6.3 (3.3 – 12.1)	.955
Total observation period in months	56.18	144.07		25.99	40.80		42.32	143.08	
Disease activity (mSLEDAI-2K score)									
1st vaccination date	2.1 ± 3.6<br>0 (0 – 4)	2.3 ± 3.6<br>(0 – 4)	.387	2.3 ± 3.4<br>4 (0 – 4)	2.4 ± 2.6<br>(0 – 4)	.426	1.5 ± 2.5<br>0 (0 – 4)	1.7 ± 2.9<br>(0 – 4)	.660
Last observation date	2.3 ± 3.6<br>0 (0 – 4)	2.3 ± 3.8<br>0 (0 – 4)	.787	2.4 ± 2.6<br>2 (0 – 4)	1.8 ± 2.00<br>0 (0 – 4)	.111	2.0 ± 3.2<br>0 (0 – 4)	1.7 ± 2.7<br>0 (0 – 4)	.423
P*-value*	0.387	0.787		0.426	0.111		0.102	0.919	
Total flares (events)	6 (9.0)	11 (16.4)	.132	2 (10.5)	5 (26.3)	.414	3 (6.5)	10 (21.7)	.034
Mild-to-moderate	1 (1.5)	7 (10.5)	.034	1 (5.3)	2 (10.5)	.564	0	5 (10.9)	.025
Severe	5 (7.5)	4 (6.00)	.564	1 (5.3)	3 (15.8)	.317	3 (6.5)	5 (10.9)	.564
Organ flares									
Mucocutaneous system (alopecia, skin rash, vasculitis rash)	0	3 (4.5)	.083	1 (5.3)	1 (5.3)	.999	0	4 (8.7)	.046
Hematological system (autoimmune hemolysis, leukopenia, thrombocytopenia)	0	2 (3.0)	.157	0	2 (10.5)	.157	0	1 (2.2)	.317
Musculoskeletal system (myositis, arthritis)	0	1 (1.5)	.317	0	0	-	0	1 (2.2)	.317
Renal system (nephritis, proteinuria)	5 (7.5)	5 (7.5)	.999	1 (5.3)	2 (10.5)	.564	3 (6.5)	3 (6.5)	.999
Neurological system (central and peripheral nervous system)	0	0	-	0	0	-	0	1 (2.2)	.317
Gastrointestinal system	0	0	-	0	0	-	0	0	-

Constitutional symptoms (fever, malaise, weight loss)	1 (1.5)	0	.314	0	0	-	0	0	-

Data are expressed as mean ± SD, median (p25 – p75), or n (%). IR (95% CI) = incidence rate (95% confidence interval) events/100 person-months.

Duration of observation and SLEDAI-2K score were analyzed by the sign rank test and paired t-test, respectively. Flares and organ flares were analyzed by McNemar's test. Incidence rate was analyzed by Poisson regression.

P-value* = *P*-value comparing the vaccination date between the 1st and 2nd doses of the same vaccine. *P*-value** = *P*-value comparing the mSLEDAI-2K score between the 1st vaccination date and the last observation date for the same vaccine

[a] = from 1st vaccine dose to 2nd vaccine dose, [b] = next visit or up to 3 months after the 2nd vaccine dose or study censor.

mSLEDAI-2K = modified Systemic Lupus Erythematosus Disease Activity Index-2000.

Supplementary Table 3. Adverse events and flares in SLE patients completing 2 doses of primary series COVID-19 vaccine (selected series)

Author, year	Country	type of study	No. of centers	No. of SLE patients	Disease activity assessment	Flare definition/ instrument	Baseline SLE disease activity	Changes in disease activity	Assessment time	[Overall AEs], {Severe} (1st, 2nd dose)	[Local AEs], {Severe} (1st, 2nd dose)	[Overall systemic AEs], {Severe} (1st, 2nd dose). Three most common organs/symptoms listed.	Flares (%), (1st, 2nd dose)
**Inactivated virus**													
Assawasaksakul T, et al. 2022	Thailand	Pro.	1	17	SLEDAI	SELENA-SLEDAI	3.1 ± 2.7	No changes in SLEDAI-2K score	4 wks after 2nd dose				No flares
Wang P, et al. 2022	China	Pro.	2	60	SLEDAI	NA	2.3 ± 3.7		46 days	[25.0%]	[1.7%]	[myalgia 6.7%; fever 5.0%, fatigue and skin rashes 3.3% each)	
Yuki EFN, et al. 2022	Brazil	Pro.	1	232	SLEDAI-2K	SLEDAI-2K	2 (0 – 19), SLEDAI-2K ≥ 6 = 18.3%	no worsening in SLEDAI-2K score	12 wks after 2nd dose	[(59.2%, 47.1%)]	[(31.8%, 27.8%)]	[(48.9%, 36.8%): headache (26.5%, 16.6%); somnolence (16.6%, 13.9%); arthralgia (15.2%, 9.9%)]	4.7%
So H, et al. 2022	Hong Kong	Pro.	1	27	SELENA-SLEDAI-2K	SELENA-SLEDAI-2K	3.0 ± 2.4	No changes in SLEDAI-2K score	4 wks after 2nd dose	[(59.3%, 63.0%)]	[(37.0%, 33.3%)]	[systemic (44.4%, 51.9%): tiredness (25.9%, 18.5%); muscle pain (14.8%, 3.7%), dizziness (11.1%, 7.4%)]	No flares. More patients had numerical improvement in SLEDAI-2K, anti-dsDNA level, and proteinuria.
Tang Q, et al. 2023	China	Retro.	1	188	SLEDAI	NA	Remission 94.7%, low disease activity 5.3%		4 wks – 3 months after 2nd dose	[(43.6%, 19.8%)]	[(14.9%, 12.1%)]	[myalgia (8.5%, 0%); fatigue (12.8%, 4.3%); headache (2.1%, 1.7%)	Severe: (0%, 0.8%)
Delkash P, et al. 2023	Iran	Cross.	1	72 (2nd dose 91.67%)	SLEDAI-2K	SLEDAI-2K	All in remission)	4 (4-4)	12 wks after 2nd dose				[19.4% (8.3%, 11.1%).

													Arthritis 12.5%, rashes 4.2%
Present study	Thailand	Retro.	1	67	mSLEDAI-2K	mSELENA-SLEDAI-2K	2.1 ± 3.6<br>0 (0 – 4)	No changes in mSLEDAI-2K score	next visit[a]	[53.7% (49.3%, 38.8%)]. {4.5%, (3.0%, 3.0%)}	[29.9% (26.9%, 23.9%)].	[consti.22.4% (20.9%, 13.4%); MSK 13.4% (10.5%, 11.9%); neurological 13.4% (13.4%, 11.9%)]	25.4% (69.00%, 16.4%). M&M: 11.9% (1.5%, 10.5%), severe 13.4% (7.5%, 6.00%)
**Adenovirus vectored**													
Assawasaksakul T, et al. 2022	Thailand	Pro.	1	28	SLEDAI	SELENA-SLEDAI	3.1 ± 3.0	No changes in SLEDAI-2K score	4 wks after 2nd dose				No flares
Present study	Thailand	Retro.	1	19	mSLEDAI-2K	mSELENA-SLEDAI-2K	3.0 ± 3.4<br>4 (0 – 4)	No changes in mSLEDAI-2K score	next visit[a]	[68.4% (68.4%, 52.6%)]. {10.5% (10.5%, 0%)}	[52.6% (47.4%, 42.1%)].	[constit. 57.9% (52.6%, 31.6%); MSK 26.3% (26.3%, 15.8%); neurological 26.3% (26.3%, 15.8%)]	36.8% (10.5%, 26.3%). M&M 15.8% (5.3%, 10.5%), severe 21.1% (5.3%, 15.8%)
**mRNA**													
Bartels LE, et al. 2021	Denmark	Pro.	1	128					1 wk after 2nd dose	[98.0%]	[85.0%]	[85.0%; fatigue 63.0%; headache 49.0%, vomiting 38.0%]	
Ferri C, et al. 2021	Italy	Pro.	21	38					2 wks after 1st and 2nd doses	[44.7%]	[39.5%]	[headache 15.8%, fever 13.2%, fatigue 2.6%]	
Moyon Q, et al. 2022	France	Pro.	1	126	SLEDAI-2K, BILAG 2004	SELENA-SLEDAI, BILAG 2004	SLEDAI-2K: 2 (0 – 4), SLEDAI-2K > 4 = 19.0%, One BILAG ≥ B = 16.7%	No changes in SLEDAI-2K score	2, 4, and 6 wks after 1st dose	[77.0%, 36.5%, and 56.5%]	[67.5%, 13.5%, 39.5]	[fatigue 39.7%, 20.6%, 35.5%; headache 25.5%, 13.5%, 39.5%; joint pain 17.5%, 8.7%, 8.1%]	2.4% increase activity (all mild), 7.14% decreased activity
Yoshida T, et al. 2022	Japan	Retro.	1	74	SLEDAI-2K	SELENA-SLEDAI	SLEDAI-2K: 0 = 18.9%, 1-5 = 24.3%, 6-10	No changes in SLEDAI-2K score	30, 60, 90 days after 2nd dose				M&M: D30, 60, 90 = 20.27%,

							= 35.1%, > 10 = 21.7%						25.68%, and 16.21%. Severe: D30, 60, 90 = 0%, 5.26%, and 8.33%
Mormile I, et al. 2022	Italy	Pro.	1	41	SLEDAI	SELENA-SLEDAI	0 = 29.27%, 1-5 = 17.07%, 6-10 = 14.63%, > 10 = 19.50%	No changes in SLEDAI score	3 wks after 1st dose, and 4 wks after 2nd dose	All. Mainly local pain, muscle pain and headache			No flares
Izmirly PM, et al. 2022	USA	Pro.	1	90	hybrid SELENA-SLEDAI	SELENA-SLEDAI	3.2 ± 3.8	No changes in SLEDAI score	2 wks after 2nd dose				11.4% M&M 10.1% Severe 1.3%
So H. et al. 2022	Hong Kong	Pro.	1	38	SELENA-SLEDAI-2K	SELENA-SLEDAI-2K	2.8 ± 1.7	No changes in SLEDAI-2K score	4 wks after 2nd dose	[(97.4%, 89.5%)]	[(94.7%, 89.5%)]	[(81.7%, 71.0%): tiredness (57.9%, 50.0%); muscle pain (44.7%, 36.8%); joint pain (18.4%, 7.9%)]	No flares. More patients had numerical improvement in SLEDAI-2K, anti-dsDNA level, and proteinuria.
Zavala-Flores E, et al. 2022	Peru	Retro.	single	100 (2nd dose 90.0%)		Increase in disease activity or laboratory in one organ			10 days after 1st and 2nd dose	[(90.0%, 92.2%)]	[(87.0%, 83.2%]. {(13.0%, 14.4%)}	[headache (45.0%, 37.8%); fatigue (39.0%, 40.3%); joint pain (29.0%, 24.5%)]. {headache (8.0%, 8.9%); fatigue 8.0%, 10.0%); joint pain (5.0%, 5.6%)}	(9.00%, 20.00%), mainly arthritis and skin
Ma M, et al. 2023	Singapore	Retro.	8	641 (2nd dose 97.3%)		New symptoms requiring treatment with CS, IMD, or hospitalization			12 wks after 1st dose				7.96% within 3 months, 3.28% after 3 months, 11.39% improved

Kikuchi J, et al. 2024	Japan	Pro.	1	90	SLEDAI-2K	SLELENA-SLEDAI	2.0 (0 – 2)	Significantly increased SLEDAI score after 2nd dose	next visit after 2nd dose	[88.9%]	[73.6%]	[79.2%; fatigue 52.8%, muscle ache 49.3%, fever 43.1%]	M&M 10.0%, severe 4.4%
Present study	Thailand	Retro	1	46	mSLEDAI-2K	mSELENA-SLEDAI-2K	1.5 ± 2.5<br>0 (0 – 4)	No changes in mSLEDAI-2K score	next visit[a]	[80.4% (67.4%, 76.1%)]. {4.6% (2.3%, 4.7%)}	[69.6% (58.7%, 63.0%)].	[constit. 37.0% (19.6%, 32.6%); MSK 15.2% (13.0%, 10.9%); neurological 17.4% (13.0%, 13.0%)]	28.3% (6.5%, 21.7%). M&M 10.9% (0%, 10.9%), severe: 17.4% (6.5%, 10.9%)

[a] = up to 3 months after the 2nd vaccine dose or study censor.

Cross. = Cross-sectional study, Retro. = Retrospective study, Pro. = prospective study.

AEs = adverse events, BILAG = British Isles Lupus Assessment Group, constit. = constitutional symptoms, MSK = musculoskeletal system, M&M = mild-to-moderate, SLEDAI = Systemic Lupus erythematosus disease activity index, SLEDAI-2K = Systemic Lupus Erythematosus Disease Activity Index – 2000, SELENA-SLEDAI = Safety of Estrogens in Lupus Erythematosus: National Assessment version of the Systemic Lupus Erythematosus Disease Activity Index, mSLEDAI-2K = modified Systemic Lupus Erythematosus Disease Activity Index – 2000, mSELENA-SLEDAI-2K = modified Safety of Estrogens in Lupus Erythematosus: National Assessment version of the Systemic Lupus Erythematosus Disease Activity Index – 2000.

Assawasaksakul T, et al. Vaccines (Basel) 2022;10:853.
Delkash P, et al. Arch Clin Infect Dis 2023;18:e139989.
Ferri C, et al. J Autoimmun 2021;125:102744.
Izmirly PM, et al. Arthritis Rheumatol 2022;74:284-294.
Ma M, et al. J Autoimmun 2023;134:102959.
Mormile I, et al. Vaccines (Basel) 2022;10:1221.
Moyon Q, et al. Ann Rheum Dis 2022;81:575-583.
So H, et al. Ther Adv Musculoskelet Dis 2022;14:1759720x221089586.
Tang Q, et al. Clin Exp Med 2023;23:457-463.
Wang P, et al. Biomed Pharmacother 2022;150:112997.
Yoshida T, et al. Lupus Sci Med 2022;9:e000727.
Yuki EFN, et al. Arthritis Care Res (Hoboken) 2022;74:562-571.
Zavala-Flores E, et al. 2022;41:1349-1357.
